# Acetic-acid-induced jasmonate signaling in root enhances drought avoidance in rice

**DOI:** 10.1038/s41598-021-85355-7

**Published:** 2021-03-18

**Authors:** Daisuke Ogawa, Yuya Suzuki, Takayuki Yokoo, Etsuko Katoh, Miyu Teruya, Masayuki Muramatsu, Jian Feng Ma, Yuri Yoshida, Shunsaku Isaji, Yuko Ogo, Mitsue Miyao, Jong-Myong Kim, Mikiko Kojima, Yumiko Takebayashi, Hitoshi Sakakibara, Shin Takeda, Kazunori Okada, Naoki Mori, Motoaki Seki, Yoshiki Habu

**Affiliations:** 1 Institute of Agrobiological Sciences, National Agriculture and Food Research Organization, Tsukuba, 305-8602 Japan; 2 Institute of Crop Science, National Agriculture and Food Research Organization, Tsukuba, 305-8517 Japan; 3 Advanced Analysis Center, National Agriculture and Food Research Organization, Tsukuba, 305-8517 Japan; 4 Biotechnology Research Center, The University of Tokyo, Tokyo, 113-8657 Japan; 5 Institute of Plant Science and Resources, Okayama University, Kurashiki, 710-0046 Japan; 6 Graduate School of Agriculture, Kyoto University, Kyoto, 606-8502 Japan; 7 Graduate School of Agricultural Science, Tohoku University, Sendai, 980-8572 Japan; 8 Plant Genomic Network Research Team, RIKEN Center for Sustainable Resource Science, Yokohama, 230-0045 Japan; 9 Graduate School of Agricultural and Life Sciences, The University of Tokyo, Tokyo, 113-8657 Japan; 10 Mass Spectrometry and Microscopy Unit, RIKEN Center for Sustainable Resource Science, Yokohama, 230-0045 Japan; 11 Graduate School of Bioagricultural Sciences, Nagoya University, Nagoya, 464-8601 Japan; 12 Bioscience and Biotechnology Center, Nagoya University, Nagoya, 464-8601 Japan; 13 Plant Epigenome Regulation Laboratory, RIKEN Cluster for Pioneering Research, Wako, 351-0198 Japan; 14 Graduate School of Life and Environmental Sciences, University of Tsukuba, Tsukuba, 305-8577 Japan

**Keywords:** Abiotic, Drought

## Abstract

Conferring drought resistant traits to crops is one of the major aims of current breeding programs in response to global climate changes. We previously showed that exogenous application of acetic acid to roots of various plants could induce increased survivability under subsequent drought stress conditions, but details of the metabolism of exogenously applied acetic acid, and the nature of signals induced by its application, have not been unveiled. In this study, we show that rice rapidly induces jasmonate signaling upon application of acetic acid, resulting in physiological changes similar to those seen under drought. The major metabolite of the exogenously applied acetic acid in xylem sap was determined as glutamine—a common and abundant component of xylem sap—indicating that acetic acid is not the direct agent inducing the observed physiological responses in shoots. Expression of drought-responsive genes in shoot under subsequent drought conditions was attenuated by acetic acid treatment. These data suggest that acetic acid activates root-to-shoot jasmonate signals that partially overlap with those induced by drought, thereby conferring an acclimated state on shoots prior to subsequent drought.

## Introduction

Drought is an abiotic stress that occurs irregularly and often causes devastating damage to crop production^1^. Strategies against drought must be considered with respect to crop habitats and climate^2,3^. Drought tolerance, which is defined as the ability to maintain normal or minimum growth with lowered water potential in the tissues^3^, is a desirable characteristic of crops cultivated in arid areas. In contrast, if drought is severe or long-lasting, drought avoidance or resilience rather than tolerance would be preferred since water shortage would overwhelm the ability of crop’s tolerance to maintain their minimum activity of life. Under such harsh conditions, plants actively retard growth, restarting when environmental conditions return to normal^4–6^. Compared with the seasonal cycle of growth retardation and restarting in perennial plants, which is a relatively slow process regulated by gradual and cumulative changes in combined environmental cues, responses to acute stresses must be rapid and accompanied with the ability to resume growth quickly when the environment recovers^6^. Understanding the molecular mechanisms of the rapid responses in crops under harsh conditions, and how to manipulate these responses, are amongst the most important challenges facing current agriculture under the global climate changes.

Plant hormones are pivotal in regulation of environmental stress responses and abscisic acid (ABA) is the major player in responses to drought, salt, and cold stresses^7^. ABA is also involved in seed dormancy, which would seem to be indicative of a possible mechanistic link in energy allocation between the seasonal cycle and acute response to environmental cues. Jasmonic acid and its related compounds (collectively termed as jasmonate, JA) is another plant hormone that has been studied intensively in terms of responses to herbivore attack^8^, and its involvement in abiotic stress responses has recently been revealed in *Arabidopsis thaliana* and other plants^9^. The physiologically active forms of JA are their amino acid conjugates, especially with isoleucine (JA-Ile), and the molecular basis of their biosynthesis and signaling has been a hot topic in recent plant molecular physiology^8^. Induction of JA production upon perception of abiotic stress has been reported, and exogenous application of JA has been shown to confer abiotic stresses tolerance in many plants^10^. However, the complex interactions of JA and other phytohormones under various abiotic stress conditions obscure the molecular mechanisms of JA-mediated abiotic stress responses.

Chromatin modification is a mechanism for regulating gene activity, and histone modifications have been shown to be rapid and reversible switches responding to environmental cues^11,12^. HDA6 in *Arabidopsis thaliana* is an epigenetic regulator known to repress transposons and maintain the structure of nucleolar organizing regions, possibly through heterochromatin formation^13,14^. Unexpectedly, we found recently that *hda6* mutants exhibited improved survival under drought conditions^15^. *hda6* mutants enhance expression of genes involved in acetic acid biosynthesis under drought conditions and accumulate acetic acid in the plant body. Treatment of wild-type *Arabidopsis* plants with exogenous acetic acid resulted in improved survival under subsequent drought conditions. The data suggest that acetic acid is the key regulator for improved survival of *hda6* under drought. Notably, the effect of acetic acid on improved survival under drought was also observed in other plants, including rapeseed, wheat, maize and rice^15^. However, it remains to be elucidated how acetic acid functions in plants. To explore the possibility of using acetic acid to enhance crop productivity, we analyzed here the physiological and molecular mechanisms of acetic-acid-mediated improvement of plant survival under drought by using rice as a model cereal.

## Results

### Acetic acid induces physiological responses similar to those induced by drought stress in rice

**Figure 1:**
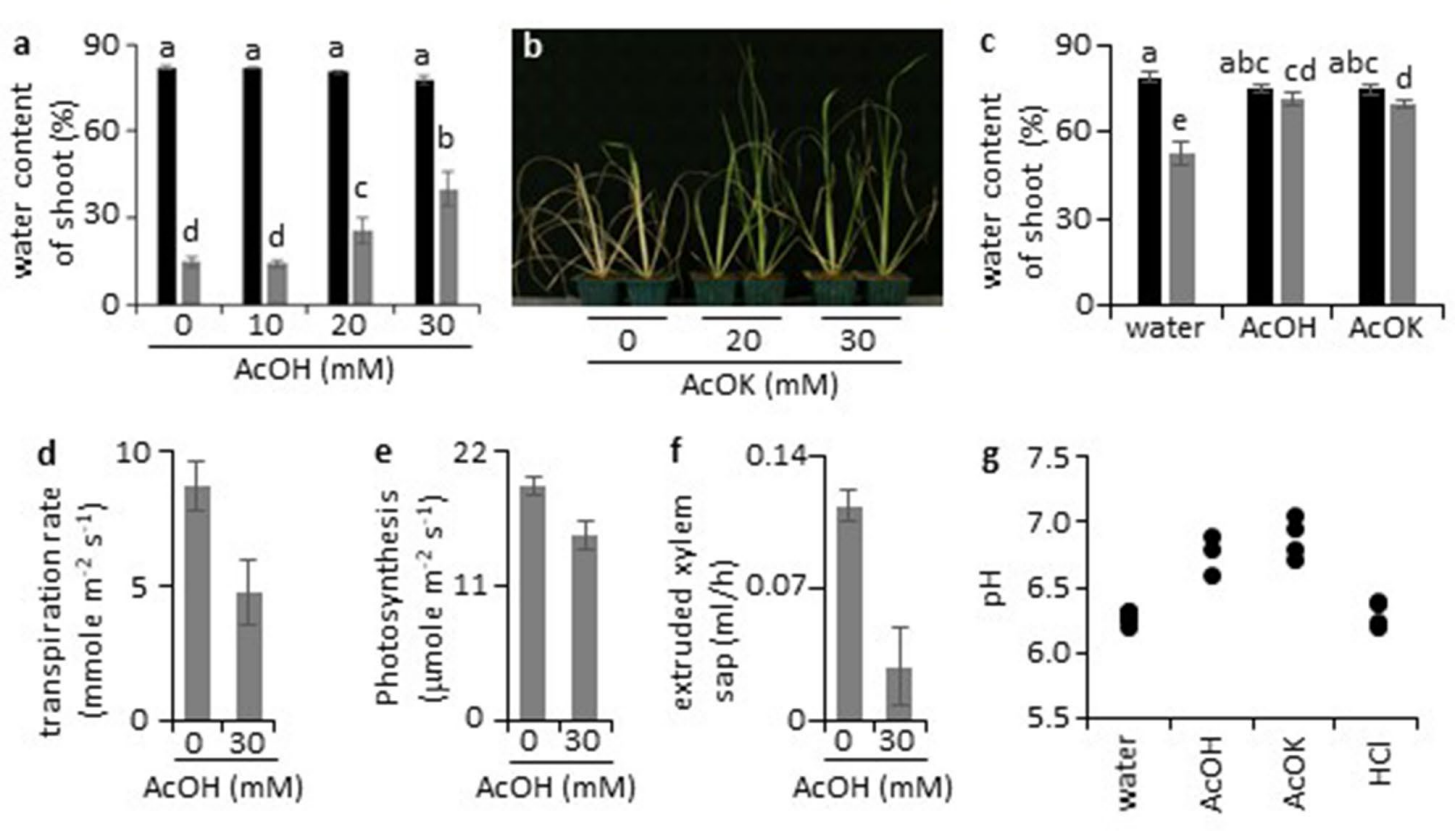
Acetic acid induces physiological changes resembling responses to drought in rice. (**a**) Water content of shoots treated with acetic acid under drought conditions. Two-week-old rice plant roots were treated with acetic acid (AcOH) solutions for 4 days; plants were then subjected to drought stress for a further 4 days. Statistical significance was examined by the Tukey Kramer method (p < 0.01). Black bars, before drought exposure; gray, day 4 of drought conditions; error bars, mean ± SD; *n* = 5 or 6. (**b**) Drought avoidance of rice induced by potassium acetate. Two-week-old rice plants were treated with potassium acetate (AcOK) for 4 days and then grown under drought conditions for 4 days. After drought treatment, plants were rewatered and grown for 4 days. (**c**) Water content of plants treated with acetic acid or potassium acetate. Water content of shoot of acetic acid or acetate-treated rice plants was measured before and after 2-day drought exposure. Statistical significance was examined by Tukey’s method (p < 0.01). Black bars, day 0; gray, day 2; error bars, mean ± SD; *n* = 8. (**d–f**) Physiological changes in acetic acid-treated rice plants. Two-week-old rice plants were treated with 0 or 30 mM acetic acid for 2 days. Transpiration rate (**d**), photosynthesis activity (**e**), and flow rate of xylem sap (**f**) are shown. Statistical significance was examined by Student’s t-test (p < 0.01). Mean ± SD; *n* = 4. (**g**) Xylem sap pH of rice plants treated with water, 30 mM acetic acid, 30 mM potassium acetate and 30 mM hydrochloric acid (HCl) for 2 days.

In our previous study, we showed that acetic acid applied exogenously to roots conferred drought tolerance to various plants including a representative temperate japonica cultivar, Nipponbare^15^. Drought tolerance following acetic acid pre-treatment was also observed in an indica (IR64) and an African upland race rice variety^16^ (NERICA1; Supplementary Fig. S1 online), indicating that the mechanism of the drought tolerance induced by acetic acid is conserved irrespective of the adaptive traits of cultivated rice. Shoot water content was around 80% in both control and acetic acid-treated plants at day 4 of acetic acid treatment (Fig. 1a). However, after subsequent drought treatment for 4 days, shoot water content decreased drastically in control plants and plants treated with 10 mM acetic acid, whereas those treated with 20 or 30 mM acetic acid retained a significantly higher water content (Fig. 1a), indicating that acetic acid treatment confers characteristics of drought avoidance on the treated seedlings^3^. Potassium acetate also induced similar drought avoidance in rice; therefore, it is likely that the ionic form of acetate is the effector inducing drought avoidance in rice (Fig. 1b,c). The effectiveness of potassium acetate in conferring improved survivability under drought excludes the possibility that the acidic pH of acetic acid is the causative agent (Fig. 1b and Supplementary Figs. S1d and S2 online).

Analysing rice seedlings during treatment indicated that acetic acid induces physiological changes in shoots that are similar to those induced by drought stress, i.e., significant decreases in transpiration rate, photosynthetic activity, and flow rate of xylem sap (Fig. 1d–f). Alkalization of xylem sap, which is a common characteristic response of plants to drought conditions^17^, was observed in seedlings treated with acetic acid (Fig. 1g). This alkalization of xylem sap was not observed in plants treated with hydrochloric acid, which does not confer improved survival under drought (Supplementary Fig. S1d online), further highlighting the correlation between the pseudo-drought responses to acetic acid treatment and improved survival in subsequent drought conditions.

To gain insight into the nature of drought avoidance of rice induced by acetic acid, we examined conditions of the treatment required for conferring drought avoidance. Rice seedlings treated with acetic acid for 1–4 days were examined for their survivability under subsequent drought. An acetic acid treatment of at least for 3 days duration was required to confer drought avoidance (Supplementary Fig. S3a online). The effect of acetic acid treatment on drought avoidance was canceled by the insertion of water treatment (Supplementary Fig. S3b online). A 1- or 2-day-water break weakened drought avoidance and a 3-day-water break abolished it. The results indicate the effect of acetic acid on rice drought avoidance is both cumulative and transient.

### Acetic acid is metabolized to glutamine and transported from root to shoot in rice

**Figure 2:**
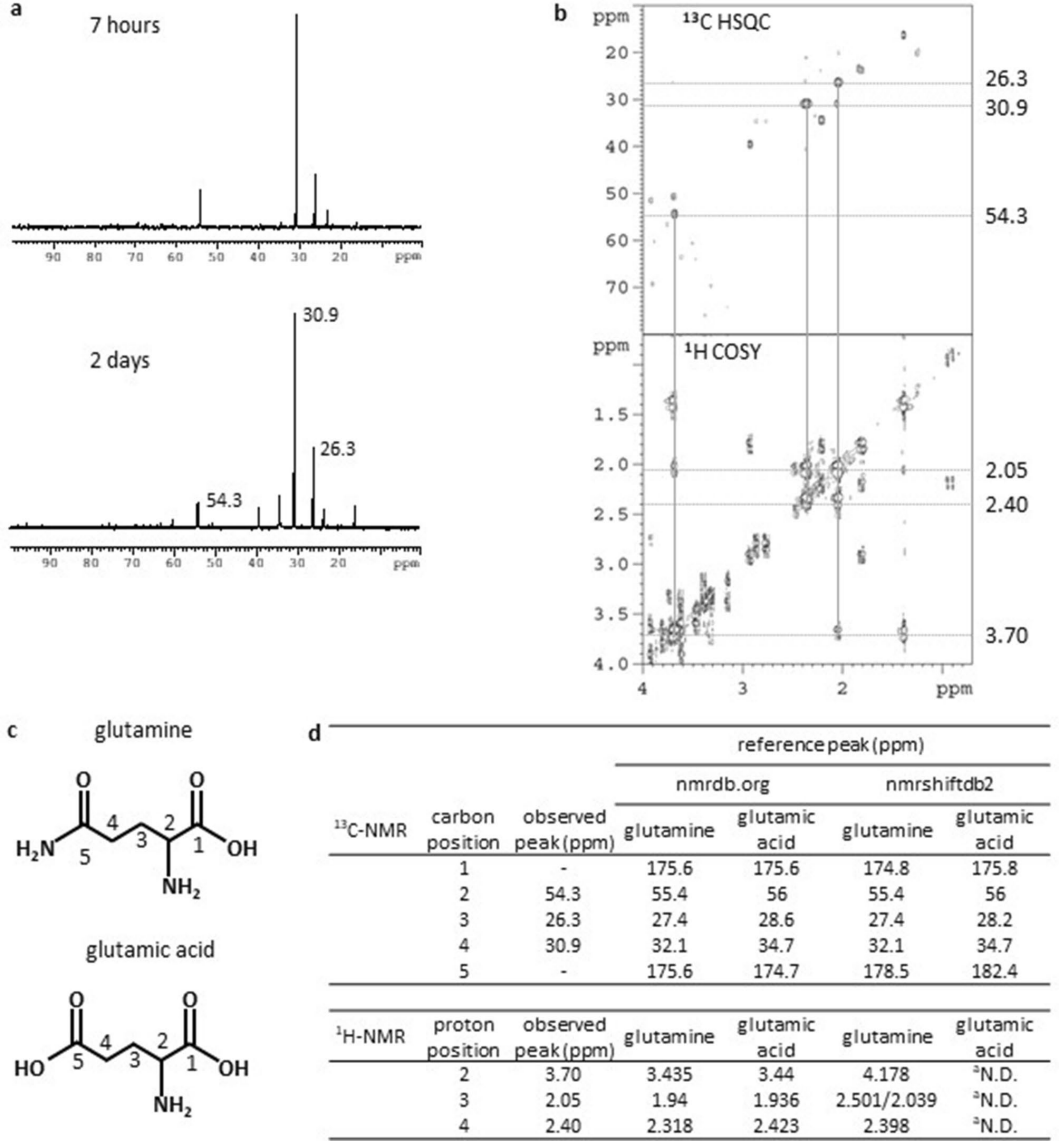
^13^C-NMR analysis of xylem sap in acetic acid-treated rice plants. (**a**) ^13^C-NMR spectra of xylem sap. Two-week-old rice plants were treated with 30 mM methyl-^13^C-labeled acetic acid for 7 h or 2 days. Xylem sap was collected and analyzed by ^13^C-NMR. Peaks in spectra observed in ^13^C-labeled acetic-acid-treated xylem sap were subtracted from those of non-labeled acetic-acid-treated xylem sap and are shown. Chemical shifts of major peaks are indicated in ppm. (**b**) 2D ^13^C-HSQC-^1^H-COSY spectra of xylem sap collected 2 days after acetic acid treatment. Chemical shifts of major peaks are shown in the right in ppm. (**c**) Structures of glutamine and glutamic acid. Positions of carbons assigned in (**d)** are shown. (**d**) Summary of observed chemical shifts and reference data of glutamine and glutamic acid. Observed chemical shifts were assigned in nmrdb.org (https://www.nmrdb.org/) and nmrshiftdb2 (https://nmrshiftdb.nmr.uni-koeln.de/). Reference data of chemical shifts in ^13^C- and ^1^H-NMR of glutamine and glutamic acid in the database are shown. ^a^No data available.

The physiological changes observed in shoots of acetic-acid-treated rice seedlings are similar to known drought responses (Fig. 1) suggest that acetic acid-induced signals in roots are transported to shoots. We have recently shown that the exogenously applied acetic acid to rice seedlings is converted to γ-amino butyric acid (GABA) in roots^18^, and therefore we examined here whether GABA and/or other metabolites are transported from roots to shoots in rice. Methyl-^13^C-labeled acetic acid was applied to 2-week-old rice seedlings and xylem sap of the treated seedlings was collected and analyzed by ^13^C-NMR (Fig. 2). At 7 h after application of methyl-^13^C-labeled acetic acid, a major peak was detected at 30.9 ppm, which corresponds to carbon 4 of glutamine. No peak for methyl-carbon of acetic acid (20.5 ppm) was observed. The presence of minor peaks at 26.3 and 54.3 ppm—corresponding to carbons 3 and 2 of glutamine—suggests that the ^13^C is also incorporated into glutamine after two or more rounds of the TCA cycle (Supplementary Fig. S4 online) or via other pathways. These assumptions were confirmed by 2D ^13^C-HSQC-^1^H-COSY spectra of xylem sap (Fig. 2b–d). The results suggest that exogenously applied acetic acid is metabolized to glutamine in root and transported to shoot through xylem. Concentration of amino acids in xylem sap was globally increased in seedlings treated with acetic acid, probably due to a reduction in the flow rate of xylem sap and subsequent concentration of solutes (Supplementary Table S1 online). Glutamine is an abundant amino acid in xylem sap, serving to transport ammonium from root to shoot^19,20^, and its concentration in xylem sap was not significantly changed in acetic-acid-treated seedlings (Supplementary Table S1 online), suggesting that exogenously applied acetic acid is incorporated into mainstream ammonium assimilation in rice and is not the direct agent inducing the physiological changes observed in shoots.

### Activation of jasmonate signaling in root by acetic acid treatment

**Figure 3:**
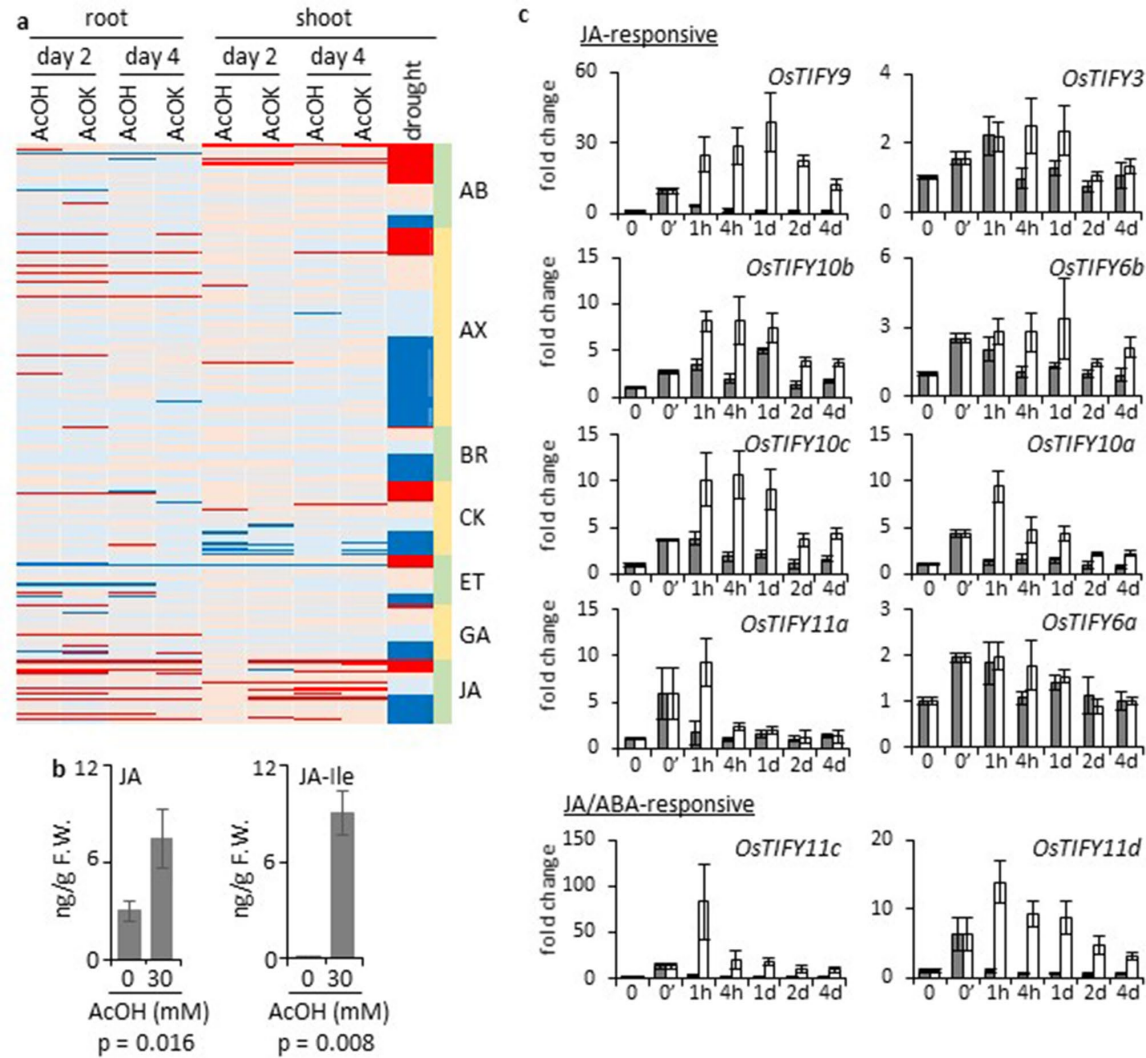
Jasmonate signaling is activated by acetic acid treatment of rice. (**a**) Microarray analysis was performed with RNA samples obtained from roots and shoots of rice seedlings treated with acetic acid (AcOH) or potassium acetate (AcOK) on day 2 and day 4 of the treatment. Data obtained with RNA prepared from shoots of rice seedlings under drought conditions was used for comparison. Changes in expression of genes involved in biosynthesis, signaling, or downstream effects of hormones [abscisic acid (AB), auxin (AX), brassinosteroid (BR), cytokinin (CK), ethylene (ET), gibberellin (GA), and jasmonic acid (JA)] are visualized as a heat-map (red; fold change ≥ 2, pale red; 1 < fold change < 2, pale blue; 0.5 < fold change < 1, blue; fold change ≤ 0.5). Genes related to each hormone were ordered according to their fold change under drought conditions. Details of genes and fold changes are shown in Supplementary Tables S2–S8 online. (**b**) Quantification of JA in acetic-acid-treated rice roots (30 min after application of acetic acid). Left, jasmonic acid; right, JA-Ile. Error bars, standard deviation; *n* = 6. Statistical significance was examined by t-test. F.W., fresh weight. (**c**) Expression analysis of *TIFY*/*JAZ* genes in acetic-acid-treated rice root by qRT-PCR. Two-week-old rice seedlings were treated with 30 mM acetic acid and total RNA was extracted from roots at the timepoint indicated (up to 4 days). Genes were classified as JA- or JA/ABA-responsive according to data in public databases (Supplementary Table S12 online). Gray bars, control water treatment; white bars, acetic acid treatment; 0, plants without treatment; 0′, plants after water draining; vertical axes, fold changes. Error bars, standard deviation; *n* = 3.

Our previous study indicated that acetic acid treatment of *Arabidopsis thaliana* induces transient production of JA, but activation of subsequent JA signaling is not observed until the treated plants are exposed to drought, indicating that the effect of acetic acid on *A. thaliana* is not a direct induction of JA-mediated drought tolerance but rather it primes JA signaling for enhanced response to subsequent drought stress^15^. We performed microarray analysis of gene expression in roots and shoots of acetic-acid-treated rice seedlings, and expression of genes related to various plant hormones was compared to that induced by drought without acetic acid treatment (Fig. 3a and Supplementary Tables S2–8 online). Consistent with the physiological changes induced in shoots of acetic-acid-treated rice plants (Fig. 1), activation of genes involved in ABA biosynthesis and its downstream signaling were observed in shoots of acetic-acid-treated plants. In fact, the acetic acid treatment triggered ABA accumulation in root and shoot (Supplementary Fig. S5 online). However, the profile of the activated genes was distinct from that induced by drought: many genes strongly activated by drought remained in an uninduced state in acetic-acid-treated plants (Supplementary Table S2 online). Activation of genes involved in JA biosynthesis and its signaling was also observed in shoots of acetic-acid-treated plants, but again the profile was distinct from that of drought treatment, and strong activation of JA-related genes that are repressed in drought-treated shoot was detected (Fig. 3a and Supplementary Table S8 online). In roots of acetic-acid-treated plants, most ABA-related genes remained uninduced but prominent activation of JA-related genes was detected. This suggests that acetic acid treatment activated JA signaling in roots and induced physiological changes in shoots that are partially similar to drought responses. Rapid accumulation of JA and JA-Ile in acetic-acid-treated roots supports activation of JA signaling by acetic acid treatment (Fig. 3b), whereas JA level was not substantially changed in shoot (Supplementary Fig. S6 online). Activation of genes encoding glutathione S-transferases and heat shock proteins, which respond to reactive oxygen species and denatured proteins, respectively, was observed in acetic-acid-treated rice plants, especially in root (Supplementary Tables S9 and S10 online). Genes encoding pathogenesis-related (PR) proteins were also induced by drought and acetic acid treatment, although the profiles of induced genes were not well correlated between drought and acetic acid treatments (Supplementary Table S11 online). These results support our interpretation that acetic acid treatment induces responses similar to those to drought stress through activation of JA signals.

To further characterize the JA signals induced by acetic acid treatment in root, changes in expression of genes involved in JA-signaling were investigated in detail (Fig. 3c). JASMONATE ZIM-domain (JAZ)/TIFY proteins are transcriptional repressors that regulate JA signaling^8^. JAZ/TIFY proteins are degraded upon JA production through the action of SCF^COI^^1^ complex, resulting in activation of JA-responsive downstream genes, and *JAZ/TIFY* genes are then rapidly induced to re-establish the repressor complex by feedback loops^21^. The rice genome carries at least 16 *JAZ/TIFY* genes that can be classified into JA-inducible, ABA-inducible, and JA/ABA-inducible groups^22^ (Supplementary Table S12 online). The rice 44 k microarray contains 14 out of 16 *JAZ/TIFY* genes, and upregulation in root in response to acetic acid treatment was observed in 7 of these genes (Supplementary Table S12 online). In addition to activation of JA-responsive *TIFY* genes, prominent activation was observed in *TIFY11c* and *TIFY11d*, which are JA/ABA-responsive, suggesting that acetic acid treatment activates signals commonly induced by ABA and JA in root. *TIFY8* has the unique characteristic of being ABA-responsive but not JA-responsive, and activation of *TIFY8* was observed in shoot, but not in root, of acetic-acid-treated rice plants (Supplementary Table S12 online), indicating that activation of *JAZ/TIFY* genes was caused by JA, but not ABA in root. Quantitative RT-PCR indicated that activation of the *JAZ/TIFY* genes was induced within 1 h of acetic acid treatment, which is consistent with rapid accumulation of JA upon acetic acid treatment (Fig. 3b,c). An activated state of some, but not all, of the *JAZ/TIFY* genes was maintained for at least 4 days after the onset of acetic acid treatment and this correlates with the cumulative effect of acetic acid treatment on drought avoidance (Supplementary Fig. S3 online).

Dehydration responsive element binding (DREB) proteins are a group of transcription factors that function in abiotic stress responses in both an ABA-dependent and -independent manner^23,24^ (Supplementary Table S13 online). Strong induction of two *DREB* genes (*LOC_Os08g43200* and *LOC_Os08g43210*), tandemly located on chromosome 8, was observed in roots of acetic-acid-treated rice plants. Both activated *DREB* genes are JA/ABA-inducible (Supplementary Table S13 online), supporting the above interpretation in which signals induced by JA in root are activated by acetic acid treatment.

### Activation of jasmonate signaling in root confers drought avoidance in rice

**Figure 4:**
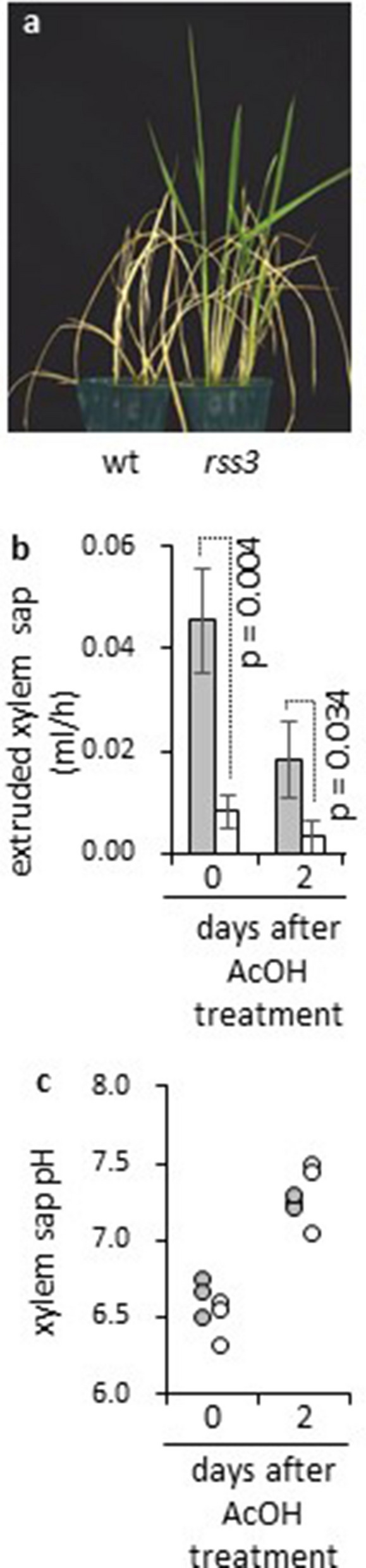
A mutant deficient in repressive regulation of JA signaling in root tip shows drought avoidance. (**a**) Wild-type and *rss3* were grown for 16 days, then subjected to drought conditions for 4 days followed by cultivation for 10 days under well-watered conditions. (**b**) Flow rate of xylem sap in wild-type (gray) and *rss3* (white) was examined before and after treatment with 30 mM acetic acid for 2 days. *n* = 3; error bars, standard deviation. Statistical significance was examined by Student’s t-test. (**c**) Xylem sap pH of wild-type (gray) and *rss3* (white) was examined before and after treatment with 30 mM acetic acid for 2 days (*n* = 3).

To further examine involvement of root JA signaling in drought avoidance of rice, we analyzed a mutant of JA signaling at the root tip. We previously reported a rice mutant showing elevated salt sensitivity, and identified the causal mutation in the *RSS3* gene encoding a putative repressor of JA signaling in the root tip^25^. Among seven *JAZ* genes activated by acetic acid in roots (Supplementary Table S12 online), five were activated in root tips of *rss3* upon salt treatment (Supplementary Table S14 online), indicating that the JA signaling pathway induced by acetic acid in roots partially overlaps that regulated by RSS3. Indeed, *rss3* showed better survival under drought without acetic acid treatment (Fig. 4a). As seen in acetic-acid-treated wild-type plants, xylem sap flow rate was reduced in *rss3* under well-watered conditions (Fig. 4b); however, no difference in xylem sap pH was observed between wild-type and *rss3* under well-watered conditions (Fig. 4c). The pH of xylem sap in *rss3* was elevated upon acetic acid treatment. Thus, alkalization of xylem sap requires signals that are distinct from those induced by the *rss3* mutation, and this property is not essential for conferring drought avoidance. Involvement of acetic-acid-induced JA signaling in drought avoidance was further confirmed by Me-JA treatment (Supplementary Fig. S7 online).

### Acetic-acid-induced signals attenuate ABA and JA signaling in shoots under subsequent drought conditions

**Figure 5:**
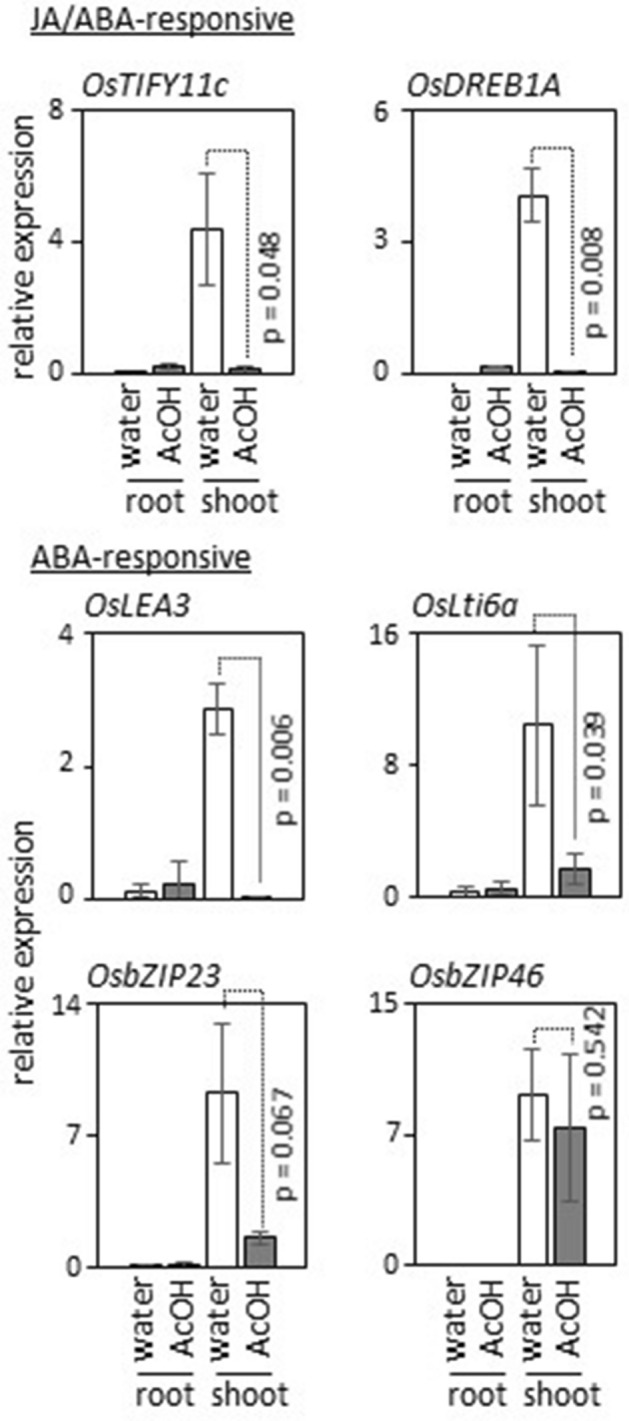
Attenuated responses of JA and ABA signaling under subsequent drought conditions. Expression levels of JA/ABA- and ABA-responsive genes in root and shoot at 1 day of drought treatment following water (white bars) or acetic acid (grey bars) treatment were analyzed by quantitative RT-PCR. Relative expression levels were calculated using *25S rRNA* as a control. *OsTIFY11c* and *OsDREB1A* are JA- and ABA-responsive genes. *OsLEA3*, *OsLti6a*, *OsbZIP23*, and *OsbZIP46* are ABA-responsive genes. Primers used are listed in Supplementary Table S17 online. Statistical significance was examined by Student’s t-test. *n* = 3.

To investigate the effect of acetic-acid-induced JA signaling on drought responses, expression of representative genes involved in ABA responses was analyzed, and the results showed that acetic acid treatment attenuated expression of most ABA-inducible genes in shoots under subsequent drought conditions^26–29^ (Fig. 5). All these genes remained in an activated state in shoots under drought without acetic acid treatment for up to at least 3 days (Supplementary Table S15 online). Among the ABA-responsive genes examined here, *OsbZIP46* was exceptional: accumulation of *OsbZIP46* mRNA was not changed significantly by acetic acid treatment, whereas expression of *OsbZIP23*, a closely related transcription factor gene, was strongly reduced compared with that of other ABA-responsive genes. Activation of *OsbZIP46* in acetic-acid-treated rice shoots under subsequent drought conditions indicated that reduced water loss in the acetic-acid-treated plants (Fig. 1a) was not the major factor for the observed attenuation of ABA-responsive genes. *OsTIFY11c* and *OsDREB1a*, which were activated by acetic acid treatment (Fig. 3; Supplementary Tables S12 and S13 online), were kept activated in roots under subsequent drought conditions, but their expression was repressed in shoots of acetic-acid-treated plants. Furthermore, no significant changes in the ABA-responsive genes examined here were detected in root of acetic-acid-treated plants (Fig. 5). The results suggest that activation of acetic-acid-induced JA signaling in root modulates ABA-responses in shoot under subsequent drought conditions and confers drought avoidance in a manner partially independent of ABA signaling. Consistent with this notion, the observed reduction in transpiration upon acetic acid treatment was attenuated in shoots of *rss3* in which a subset of JA signaling is activated in the root tip (Supplementary Fig. S8 online; Toda et al.^25^).

## Discussion

Our previous study showed that acetic acid enhances drought avoidance and that activation of JA signaling is a key event for the enhancement in *Arabidopsis*^15^. In this study, however, it appeared that the action of acetic acid on induction of JA signals is quite different in rice, indicating differences between the two model plants. Direct induction of JA-responsive genes by acetic acid treatment was not observed in *Arabidopsis*, and subsequent drought treatment was required for induction of the JA-responsive genes. These data suggest that acetic acid functions to prime JA-responsive genes in *Arabidopsis*^15,30^. The discrepancy might be ascribed to differences in culture conditions of Arabidopsis and rice, respectively. For rice, plants were grown under anaerobic conditions in soil-containing pots that were soaked in acetic acid solution (see “Methods”), whereas *Arabidopsis* plants were grown in soil under aerobic conditions^15^. Although acetic acid solution is expected to be buffered by absorbing to soil (Supplementary Fig. S2 online), large excess volumes of acetic acid solution relative to soil would be much more effective for incorporation into rice roots than was the case for *Arabidopsis* in our study.

In *Arabidopsis*, carbon atoms of exogenously applied acetic acid are incorporated into histones, possibly as acetyl groups at histone N-terminal polypeptides, and may contribute to activation of genes for drought tolerance^15^. In rice, exogenously supplied acetic acid was converted to glutamine within 7 h and transferred to shoot through xylem (Fig. 2). Since glutamine is the most abundant amino acid in rice xylem^19^ (Supplementary Table S1 online), and the concentration of glutamine in xylem sap of acetic-acid-treated rice plants is not significantly different from that of control plants (Supplementary Table S1 online), the role of exogenously applied acetic acid in induction of drought avoidance in rice would not function via histone acetylation as suggested in *Arabidopsis*. Acetic acid is incorporated into root cells as acetate anion and may induce membrane depolarization, which is also induced by herbivore attacks, and subsequently induces JA biosynthesis^31^. Elevation of potassium and sodium ions in xylem sap of acetate-treated rice plants might correlate with the disturbance of ion balance by acetate anion (Supplementary Table S16 online). Further analysis is needed towards understanding the mechanism of acetic acid-induced JA signal activation.

**Figure 6:**
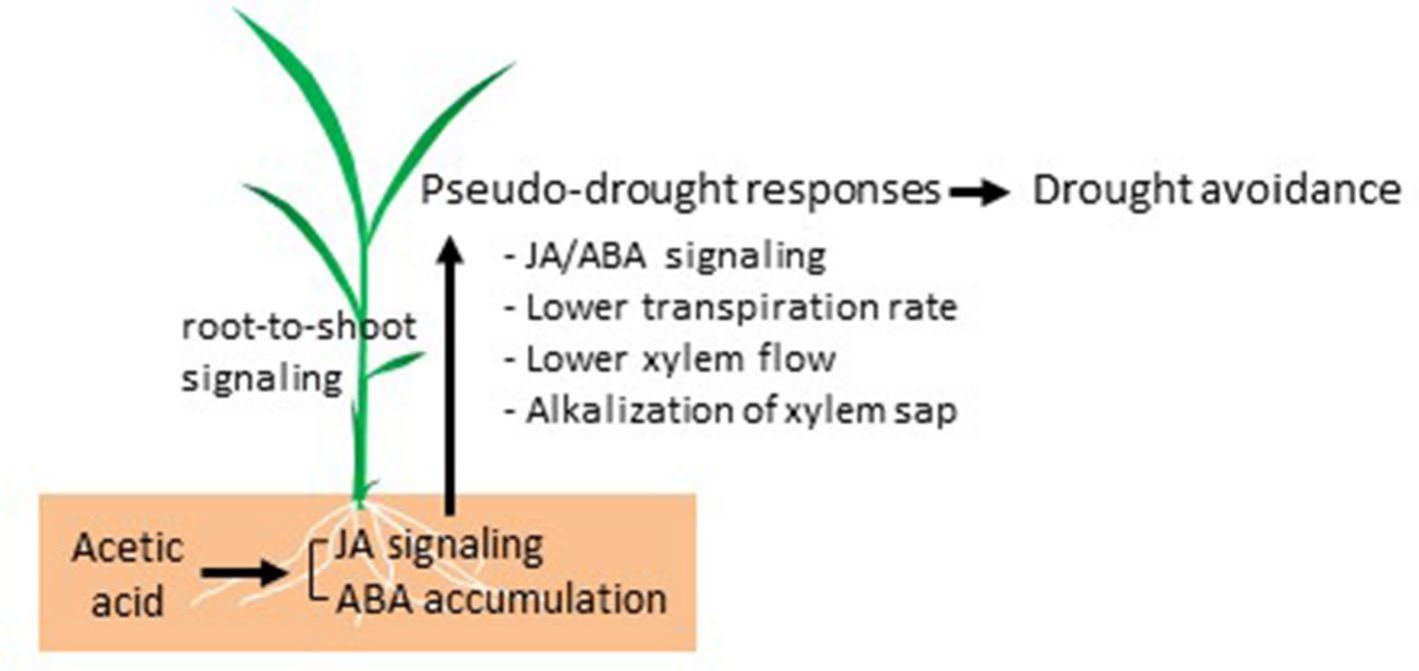
Schematic model of drought avoidance in rice plants treated with acetic acid. Acetic acid treatment of rice root induces the JA signaling and ABA accumulation in root. This event triggers pseudo-drought responses in shoot through unidentified root-to-shoot signaling. The JA/ABA signaling and induced physiological changes confer drought avoidance to rice plants.

JA production and activation of JA signaling genes were induced within 1 h of acetic acid treatment in root, but a 2-day acetic acid treatment is insufficient to confer drought avoidance in rice (Fig. 3b,c; Supplementary Fig. S3 online). We also noticed that draining water from soil before acetic acid treatment induces weak and transient JA responses in root, but again not enough to induce improved survival under subsequent drought (Fig. 3c; Supplementary Fig. S3 online). These observations could be explained by postulating cumulative effects of acetic acid on physiological or morphological changes induced during the treatment. A schematic model of the possible effects of acetic acid treatment leading to drought avoidance is shown in Fig. 6. In this model, acetic acid induces JA signaling and ABA accumulation in root and results in pseudo-drought responses including decrease of xylem flow from root to shoot and transpiration rate, and alkalization of xylem sap. At least decrease of transpiration rate could be mediated by JA/ABA signaling in shoot because JA signaling induces stomatal closure mediated by ABA^32^. Such physiological changes lead to drought avoidance. This model reminds us “priming”, The priming is a mechanism which leads to a physiological state that enables plants to tolerate to future stress triggered by pretreatment of environmental stresses and chemicals^33,34^. Under drought condition, in acetic-acid-treated plants, JA/ABA signaling was moderated compared to control plants (Fig. 5). Such weakened response to stresses is known as a feature in primed plants^35^. Signaling molecule transmitted from root to shoot for inducing the pseudo-drought response is still missing. Acetic acid itself should not be the candidate of the signaling because our experiment using methyl-^13^C-labeled acetic acid revealed the majority of acetic acid was metabolized into glutamine. Analysis of candidates for long-distance signaling, such as electrical signal^36,37^ in systemic response, conjugates of ABA^38^ and small peptides for ABA biosynthesis^39^ would provide a clue to understand the mechanism of acetic-acid-induced pseudo-drought response.

Among 20 *JAZ/TIFY* genes in the rice genome, 5 (*TIFY10b*, *10c*, *11b*, *11c*, and *11d*) were detected as commonly activated in acetic-acid-treated and *rss3* roots (Supplementary Tables S12 and S14 online). All these *JAZ/TIFY* genes are JA responsive, whereas only *TFY11c* and *TIFY11d* are responsive to both ABA and JA (Supplementary Table S12 online). It is worth noting that the two *DREB* genes strongly activated by acetic acid treatment are responsive to both ABA and JA (Supplementary Table S13 online). Considering the observed attenuation of ABA signaling under drought conditions following acetic acid treatment (Fig. 5), activation of ABA/JA-responsive *TFY11c*, *TIFY11d*, and the two *DREB* genes might act to converge JA and ABA signals and bypass drought-responsive ABA pathways. Downregulation of ABA-responsive genes observed in the root tip of *rss3* supports the antagonistic action of JA on ABA signaling in root^25^.

Suppression of ABA signaling by immune signaling has been observed in plants^40,41^, indicating that the effect of acetic acid on reduced expression of ABA-dependent genes under subsequent drought conditions could partially mimic biotic stress responses, and that acetic acid might have the effect of promoting crosstalk between biotic and abiotic stress responses. Another possibility is that acetic-acid-induced JA-signaling might reflect perception of as yet uncharacterized biotic or abiotic stimuli. In the paddy field, acetic acid (and many other organic compounds) are produced by bacteria that ferment various organic matters under anaerobic conditions. It would be beneficial to plants to utilize chemicals produced by cohabitating and symbiotic microorganisms, not only as energy sources but also as activators for promoting growth and tolerance to environmental stresses^42,43^. Low molecular weight organic compounds that exhibit beneficial effects on plant growth have been reported^44–46^ and it would be important for sustainable agriculture to address whether the effects of these various compounds on plant growth and defense is specific, or if they share common JA signaling pathways. Although the detailed mechanisms remain to be unravelled, the findings presented here already suggest that treatment with simple, easily available and low cost compounds could have hitherto unforseen beneficial effects on the growth of important crop plants.

## Methods

### Evaluation of acetic acid-induced improvement of survival under drought

Rice plants were grown in well-watered conditions (30 °C, 14 h light/10 h dark, approximately 150 ml soil/pot) for 2 weeks. Treatment of seedling roots with 30 mM acetic acid or potassium acetate and evaluation of drought avoidance were performed as described previously^15^.

### Measurement of physiological effects of acetic acid treatments

Water content of acetic acid-treated plants under subsequent drought condition (4 days) was measured by subtracting lyophilized plant weights from the corresponding fresh plant weights. Transpiration rate and photosynthetic activity of plants under acetic acid treatment were measured using a LI-6400 portable photosynthesis system (LI-COR, Lincoln, NE, USA). The flow rate of xylem sap in plants under acetic acid treatment was determined by measuring the volume of xylem sap exuded in 30 min from the shoot, cut at approximately 1 cm above the soil surface. Leaf surface temperature was measured with a Thermo Tracer (TH9100MJN, NEC, Japan).

### NMR experiments

Two-week-old plants were treated with acetic acid-2-^13^C (Sigma-Aldrich, 279,307) for 7 h or 2 days. Xylem sap was collected as described above. 1D ^13^C NMR spectra were recorded in 10% D_2_O on a Bruker Avance 500 spectrometer operating at 125.77 MHz at 25 °C. 2D COSY (homonuclear shift correlation) and ^13^C HMBC (heteronuclear multi quantum coherence) spectra were recorded in 10% D_2_O on a Bruker Avance 600 spectrometer operating at 600 and 150 MHz for ^1^H and ^13^C, respectively, at 25 °C. 2D COSY spectra were recorded with 16 transients over 128 increments (zero-filled to 1 K) and 2 K data points with spectral widths of 6009 Hz. The repetition time was 2.0 s. Phase-sensitive ^1^H-detected ^13^C-HMQC spectra were recorded with 40 transients over 200 increments (zero-filled to 1 K) and 2 K data points with spectral widths of 7 kHz in *F*2 and 15 kHz in *F*1. The repetition time was 1.0 s. The delays were adjusted according to a coupling constant 1* J* (CH) of 145 Hz. Chemical shifts are expressed in δ (ppm) values relative to TMS as external reference.

### Microarray and expression data

Acetic acid treatments were performed as described above. For drought treatment, pots containing 2-week-old plants were drained and grown without water supply for 3 days. Total RNA was extracted by RNeasy Plant Mini Kit (QIAGEN, Hilden, Germany) from shoot and root separately on day 2 and day 4 of the acetic acid treatments, or on day 3 of drought treatment without acetic acid treatment. Microarray analyses were performed as described previously^25^ with a rice 44 k microarray (Agilent GEO platform GPL6864). Raw data obtained in a microarray scanner were processed with the Limma package in Bioconductor^47^, and differentially expressed genes were detected by an empirical Bayes method equipped in Limma^4﻿8﻿^. Data for ABA- and/or JA-inducibility of *JAZ*/*TIFY* genes in rice root and shoot were retrieved from RiceXPro (http://ricexpro.dna.affrc.go.jp/) and TENOR (http://tenor.dna.affrc.go.jp/). Accession numbers of the microarray data are as follows: GSE41442 for NaCl-treated *rss3* root tip^25^; GSE115825 for acetic-acid- or potassium-acetate-treated rice shoots and roots; GSE115826 for rice shoots under drought.

### Quantification of JA and JA-Ile

Two-week-old rice plants grown on soil were treated with 30 mM acetic acid for 30 min as described above. Roots were harvested, ground to a fine powder, and subjected to extraction and quantification of jasmonates as described previously with slight modification^49^. Briefly, the concentrated sample was subjected to LC–ESI–MS/MS, which was composed of a quadrupole tandem mass spectrometer (API-3000) with an electrospray ion source and an Agilent 1100 HPLC instrument (Agilent Technologies, Palo Alto, CA, USA) equipped with a CAPCELL CORE C18 column (length 50 mm, diameter 2.1 mm; OSAKA SODA CO., LTD Osaka, Japan). The solvents used for both columns were water (A) and acetonitrile containing 0.1% (v/v) acetic acid (B), respectively. A 15-min linear gradient (3–70% B) was applied just after sample injection (flow rate 0.2 ml min^−1^). The multiple reaction-monitoring mode was used in ESI–MS/MS to monitor precursors and products. JA and JA-Ile were analyzed in the negative ion mode with nitrogen as the collision gas. JA, [^2^H_2_]-JA, JA-Ile and [^13^C_6_]-JA-Ile were determined with combinations of m/z 209/59, m/z 211/59, m/z 322/130, and m/z 328/136, respectively.

### Quantification of ABA

ABA was extracted and semi-purified as previously described^50,51^. ABA was quantified with a ultra-high performance-liquid chromatography (UHPLC)-electrospray interface-quadrupole-orbitrap mass spectrometer (UHPLC/Q-Exactive™; Thermo Scientific) as described previously^52^ with an ODS column (AQUITY UPLC BEH C18 1.7 µm, 2.1 × 100 mm, Waters).

### qRT-PCR

Total RNA was extracted with the TriPure Isolation Reagent (Roche, Basel, Switzerland) and subjected to DNA digestion by treatment with RNase-free DNase I (TAKARA Bio, Kusatsu, Japan). One microgram of total RNA was reverse-transcribed with anchored-oligo (dT) 18 primers and random hexamers using Transcriptor first-strand cDNA synthesis kit (Roche, Basel, Switzerland) and quantitative PCR analysis was performed using SYBR Premix Ex Taq II (TAKARA Bio, Kusatsu, Japan). Expression levels of target genes were estimated relative to that of 25S ribosomal RNA and shown in fold changes from those at time 0 for Fig. 3c. All assays were performed using three biological replicates. Primers used are listed in Supplementary Table S17 online.

## Supplementary Information

Supplementary Information 1.

Supplementary Information 2.

## Data Availability

The datasets generated during and/or analysed during the current study are available with the corresponding author, and can be accessed on reasonable request.
