# Supplementary material for: Acetic-acid-induced jasmonate signaling in root enhances drought avoidance in rice
Source: Sci Rep. 2021 Mar 18;11:6280. doi: 10.1038/s41598-021-85355-7 (PMC7973560; doi:10.1038/s41598-021-85355-7)
Supplement: Supplementary file 1 — Supplementary Information 1. [file 41598_2021_85355_MOESM1_ESM.pdf]

Acetic-acid-induced jasmonate signaling in root enhances drought avoidance in rice

Daisuke Ogawa, Yuya Suzuki, Takayuki Yokoo, Etsuko Katoh, Miyu Teruya, Masayuki Muramatsu, Jian Feng Ma, Yuri Yoshida, Shunsaku Isaji, Yuko Ogo, Mitsue Miyao, Jong-Myong Kim, Mikiko Kojima, Yumiko Takebayashi, Hitoshi Sakakibara, Shin Takeda, Kazunori Okada, Naoki Mori, Motoaki Seki, Yoshiki Habu

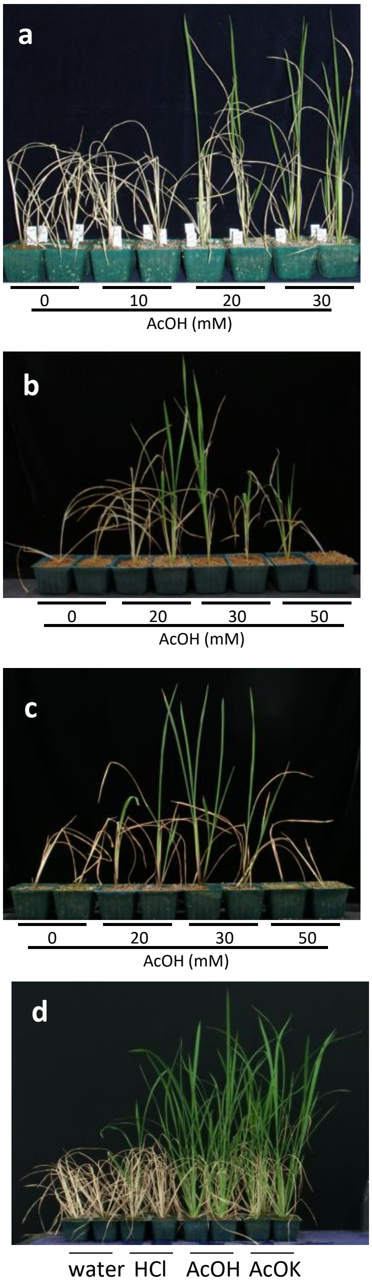

Supplementary Figure S1. Acetic acid confers drought avoidance to various rice.
Two-week-old rice plants were treated with acetic acid for 4 days, subjected to drought stress for successive 4 days, and rewatered for 10 days. **a**, Nipponbare; **b**, IR64; **c**, NERICA 1. **d**, Two-weeks-old rice plants (Nipponbare) were treated with water, 30 mM HCl, 30mM acetic acid or 30 mM potassium acetate for 4 days and then the plants were incubated under drought conditions for 4 days. After the drought treatment, plants were rewatered for 8 days.

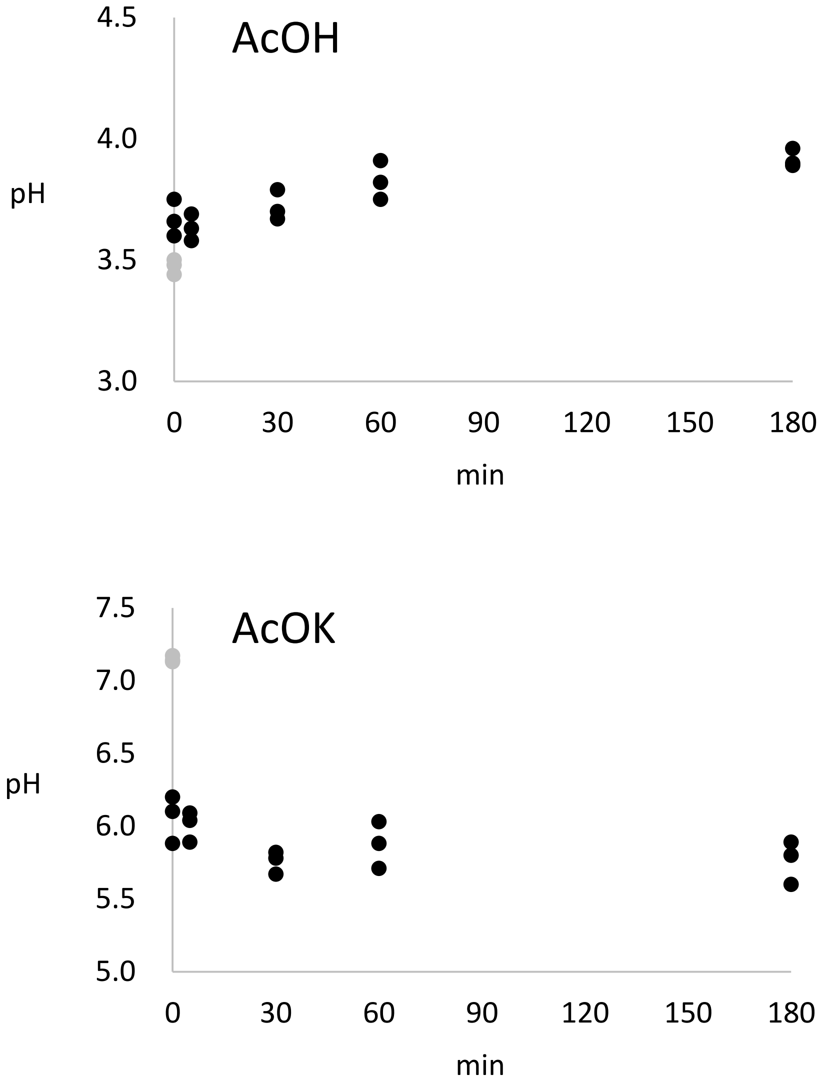

Supplementary Figure S2. Changes in pH of acetic acid and potassium acetate solutions during treatment of planted rice.

Rice plants (Nipponbare) were grown on soil (BonSol No.2, Sumitomo Chemicals, Tokyo, Japan) in square pots (6 cm x 6cm x 5cm, 1 plant per pot) for 2 weeks. Six pots were placed in a vat (21.5 cm x 8.5 cm x 5 cm) containing 1 litter of water. For acetic acid (AcOH) or potassium acetate (AcOK) treatment, pots were taken out of the vat, drained on a stack of paper towels for 20 min, and transferred to vats containing 1 litter of water, 30 mM acetic acid, or 30 mM potassium acetate. pH was measured at time points indicated in the graphs. gray, pH of 30 mM acetic acid (upper) or potassium acetate (lower) solution without soil; black, pH of 30 mM acetic acid (upper) or potassium acetate (lower) solution with soil. *n* = 3 vats.

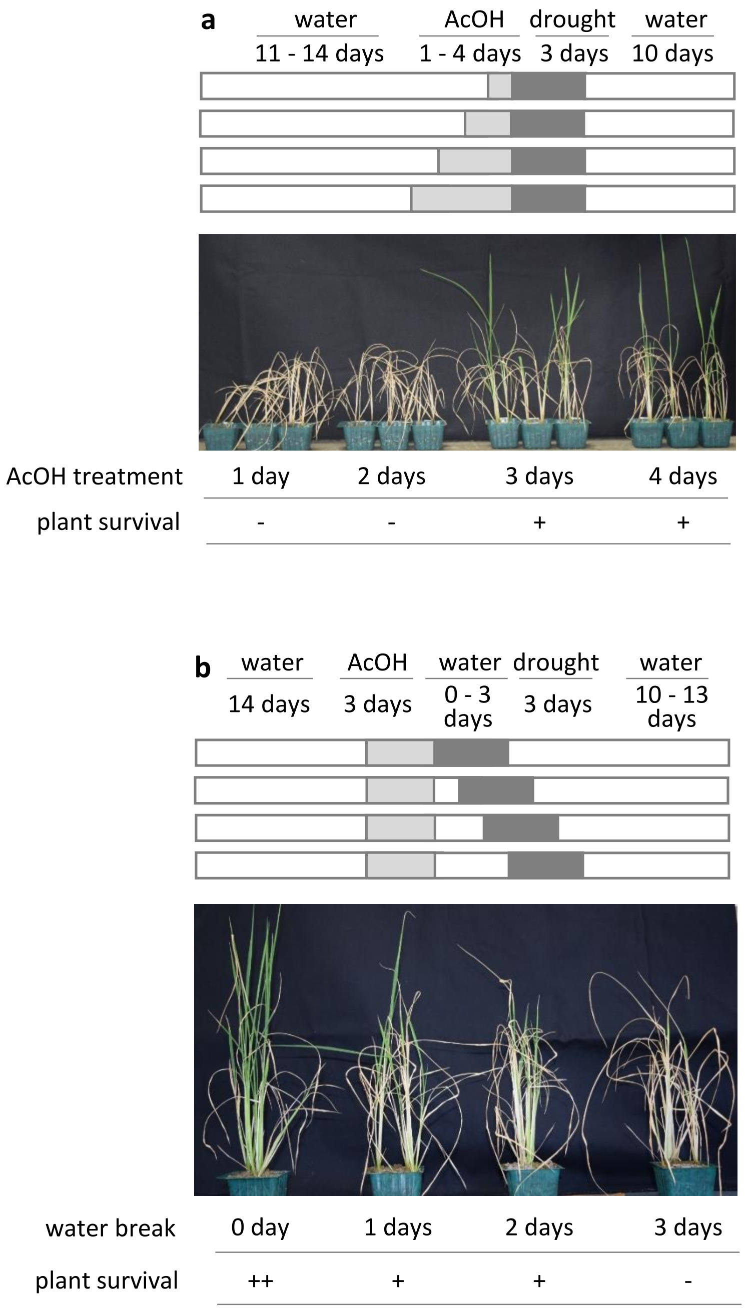

Supplementary Figure S3. Effects of acetic acid on drought avoidance is cumulative and transient.

a. Eleven- to 14-days-old rice plants were treated with 30 mM acetic acid (AcOH) for 1 to 4 days and then grown without water supply for 3 days before rewatering. Survival of treated plants were counted on 10 days after rewatering. +, survived; -, died.

b. Two-weeks-old rice plants were treated with 30 mM acetic acid for 3 days. Water was supplied to the plants at 0 to 3 days and grown without water supply for 3 days before rewatering. Survival of treated plants were counted on 10 - 13 days after rewatering. ++, vigorous; +, survived; -, died.

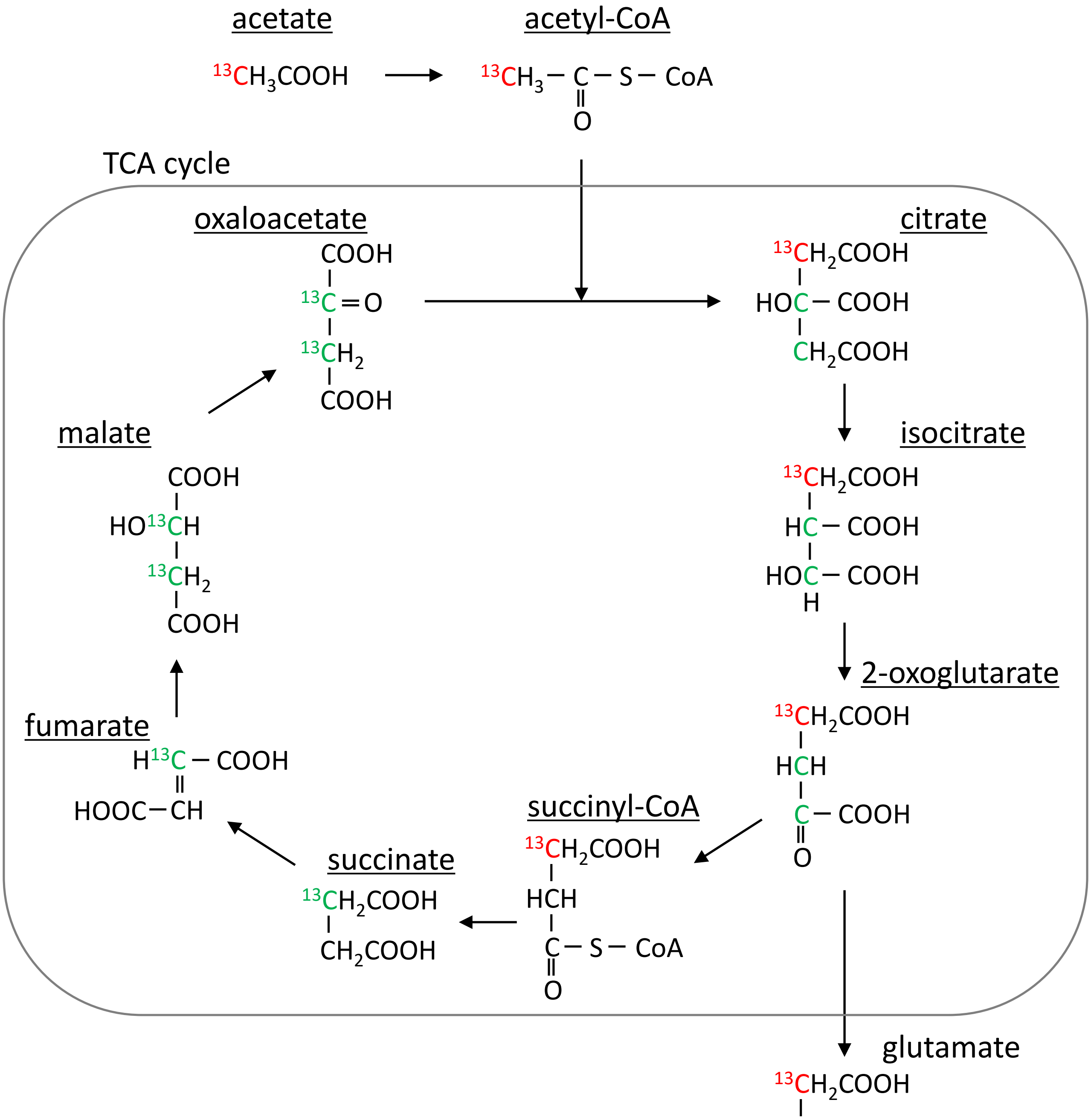

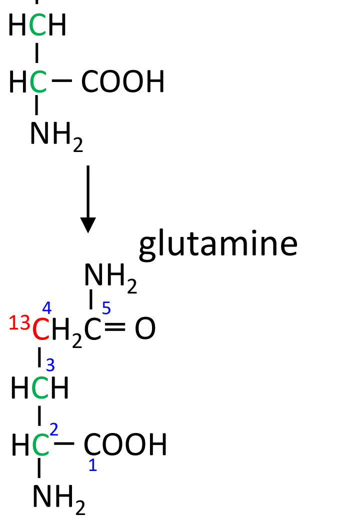

Supplementary Figure S4. Deduced metabolic pathway of exogenously applied acetic acid deduced by ^{13}C-NMR data.
^{13}C-NMR analysis of xylem sap indicated ^{13}C in methyl group of acetic acid (red) is mainly incorporated into carbon 4 of glutamine in root and transferred to shoot, and additional minor peaks corresponding to carbon 2 and carbon 3 are observed too (Figure 2). A possible pathway from acetic acid to glutamine is shown here. Note that citrate is sterically asymmetric and labeled at a fixed position. Structures of succinate and fumarate are symmetric so that ^{13}C derived from the labeled acetic acid (green) can be present either one of carbon 2 or carbon 3 in malate and oxaloacetate that are eventually incorporated into carbon 2 or carbon 3 in glutamine derived from α-ketoglutarate in the second round of TCA cycle. Positions of ^{13}C in succinate and its downstream compounds in the second and later cycles are not considered here.

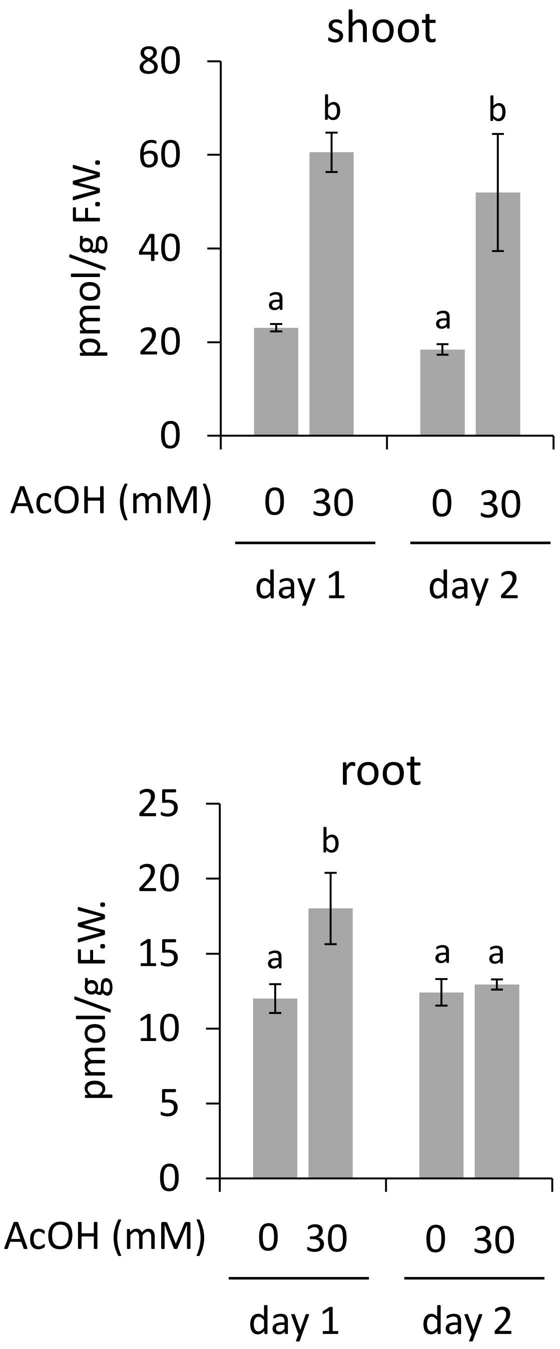

Supplementary Figure S5. Accumulation of ABA in shoot and root of acetate-treated rice plants.

Quantification of ABA in acetic-acid-treated rice shoots (days 1 and 2 after application of acetic acid). Upper, shoot; lower, root. Error bars, standard deviation; *n* = 4. Two-week-old wild-type plants were used. Statistical significance was examined by Tukey test ($p < 0.01$). F.W., fresh weight.

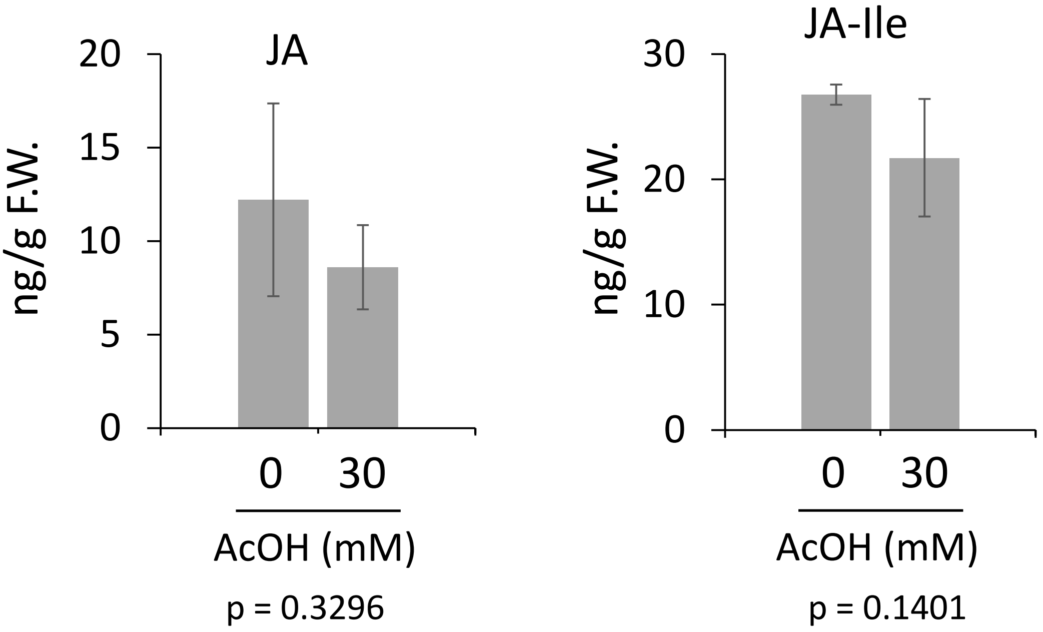

Supplementary Figure S6. Accumulation of JA and JA-Ile in shoot of acetate-treated rice plants.
Quantification of JA in acetic-acid-treated rice shoots (30 min after application of acetic acid). Left, JA; right, JA-Ile. Error bars, standard deviation; n = 6. Two-week-old wild-type plants were used. Statistical significance was examined by t-test. F.W., fresh weight.

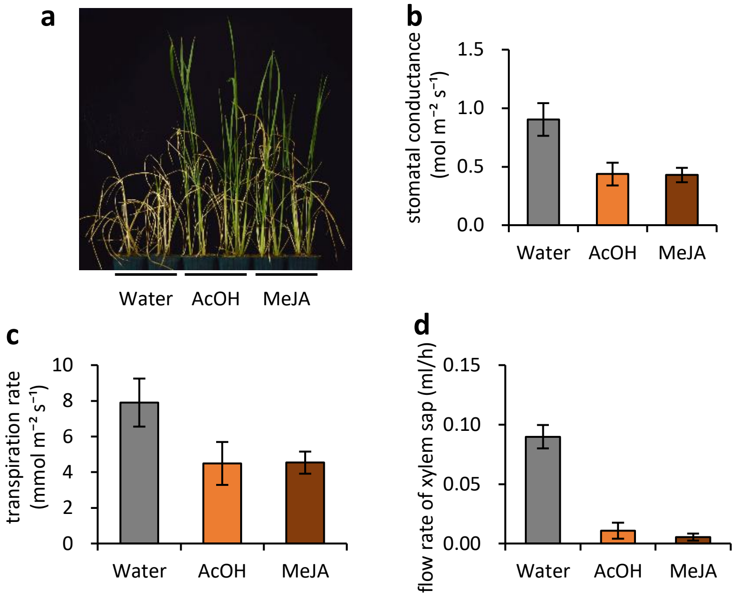

Supplementary Figure S7. Methyl-jasmonate confers drought avoidance to rice accompanied with physiological changes similar to those induced by acetic acid. Two-week-old rice plants were treated with 30 mM acetic acid (AcOH) or 100 μM methyl-jasmonate (Me-JA) for 4 days, subjected to drought stress for 4 days, and then grown under well-watered condition for 10 days. (**a**) drought avoidance of plants treated with AcOH or Me-JA, (**b-d**) stomatal conductance (**b**), transpiration rate (**c**), and (**d**) flow rate of xylem sap of plants treated with AcOH or Me-JA.

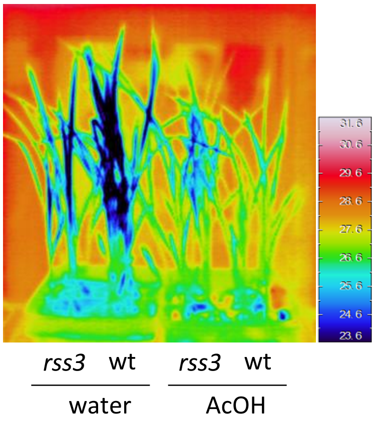

Supplementary Figure S8. Reduction in stomatal transpiration by acetic acid is attenuated in *rss3*.

Two-weeks-old wild-type and *rss3* plants were treated with (AcOH) or without (water) 30 mM acetic acid for 4 days. To monitor the rate of stomatal transpiration, leaf temperature was analyzed by infrared thermography. Temperature (°C) is indicated by color scale. Under the condition without acetic acid treatment, leaf temperature is lower in wild-type plants (wt) than *rss3*, reflecting that lower rate of stomatal transpiration in *rss3*. Note that reduction in stomatal transpiration in response to acetic acid is attenuated in *rss3*.
